# Investigation of the Relationship Between Aggression and Adult Attachment in Healthcare Professionals

**DOI:** 10.7759/cureus.19360

**Published:** 2021-11-08

**Authors:** Spyros Baras, Dimitra Lekka, Maria Bokari, Konstantina Orlandou, Vasileia Arachoviti, Aikaterini Roubi, Athanasios Tsaraklis, Argiro Pachi, Athanasios Douzenis

**Affiliations:** 1 Department of Psychiatry, Sotiria Thoracic Diseases Hospital of Athens, Athens, GRC; 2 Mental Health - Cognitive Behavior Therapy (CBT), The 417 Army Equity Fund Hospital (417 Νοσηλευτικό Ίδρυμα Μετοχικού Ταμείου Στρατού) (NIMTS), Athens, GRC; 3 Department of Psychology, Panteion University, Athens, GRC; 4 Department of Psychology, Panteion University of Social and Political Sciences, Athens, GRC; 5 Department of Dermatology, Sotiria Hospital, Athens, GRC; 6 Department of Psychiatry, Sotiria Hospital, Athens, GRC; 7 Department of Psychiatry, National and Kapodistrian University of Athens, Medical School, Athens, GRC

**Keywords:** patients, violence, health care professionals, adult attachment, aggression

## Abstract

Introduction

Multiple references to the violent and especially difficult patient have been presented by the international literature. However, there is little literature on the aggressive behaviors of health professionals in their workplaces. The aim of this research is to record and correlate aggression and attachment type data of adult health professionals.

Methods

The sample includes 192 individuals (43 men and 149 women) health professionals in the private and public sector, aged 20 to 60 years, who were selected by the method of random sampling. The survey was conducted from February 2018 to May 2018. The Greek version of the Aggression Questionnaire and the Greek version of the Revised Experiences in Close Relationships (G-ECR-R) self-report inventory were used and the analysis was performed with the Statistical Package of Social Sciences (SPSS 26) (IBM Corp., Armonk, NY).

Results

The analysis shows that the dimension of avoidance has a positive correlation with hostility and physical aggression and the dimension of stress has a positive correlation with anger, physical aggression and hostility. It also seems that the stress dimension of the adult attachment contributes significantly positively to the prediction of anger and the stress dimension contributes significantly to the prediction of hostility. The dimension of avoiding adult attachment contributes significantly to the prediction of physical aggression.

Conclusions

To our knowledge, no studies were found in the literature to examine the relationship between the subscales of aggression and dimensions of attachment. It is important that violence in the workplace is recognized as an underlying occupational risk and not just as a matter of criminal law. Finally, more research is needed to study the phenomenon in order to make it more understandable.

## Introduction

Webster et al. [1] defined aggression as the obvious behavior, which includes the clear intention to cause painful stimuli to another organism or to manifest destructive behavior towards inanimate objects. They distinguished it, in verbal aggression (hostility), in physical aggression against objects (destructiveness), in physical aggression against other people (harmful - violence). Impulsive aggression is defined as a reaction to a perceived challenge to an individual's attempt to defend oneself from a stimulus perceived as threatening and accompanied by some visible forms of anger [2]. Premeditated aggression is defined as an attempt by a person to influence or force another person to do something without being challenged. It is more focused on one goal than impulsive aggression [2].

Peek-Asa et al. [3] have described the four categories of violence:

Type I (Criminal Intent): The perpetrator has nothing to do with the workplace and commits a criminal act (e.g., robbery).

Type II (client to employee): The perpetrator is a client of the services/products of the workplace (e.g., patient, attendant, student, prisoner).

Type III (employee to employee): The perpetrator and the victim are co-workers or former co-workers in the same workplace.

Type IV (Personal Relationship): The perpetrator usually has a personal relationship with an employee (e.g., an incident of domestic violence that takes place in the workplace of the perpetrator or the victim).

The type III violence (employee-to-employee violence) involves a wide range of events, such as physical assault, verbal assault, harassment, threats, and bullying. Τhis in the workplace constitutes mobbing. Thus, younger nurses may be intimidated by senior nurses [4], physicians by nurse practitioners [5] and nurses may be targeted by physicians [6]. The type III violence can also involve bodily harm, but it always involves a psychological component that can be detrimental to employee well-being [7-9].

The attachment theory is a way of understanding the tendency of individuals to build strong emotional attachments with other people and to understand the various forms of emotional distress (e.g., anger, despair, rejection). According to the theory, the early experiences with caregivers are expressed in "standard attachment behaviors" during development and create multiple internal representations or Internal Working Models (IWMs) of self and others, which are activated in adults when they are exposed to threats. Over time, IWMs produce unique patterns of thoughts, feelings, and behaviors that reflect different types of adult attachment. When an adult is exposed to a real or symbolic threat, the system is activated and he or she feels the desire to seek and restore closeness (real or symbolic) with an attached person. When the safety goal is achieved, the attachment system is deactivated and the individual can return peacefully to non-binding activities [10].

Research findings show that the safe type is positively associated with emotional intelligence [11]. The responses of individuals categorized as "Reject-avoidance" are characterized by an inability to recall specific examples in parenting, to idealize parents, and to downplay the importance of these relationships to oneself. Preoccupied individuals are characterized by intertwined habits and patterns (confused as to whether they have a positive or negative view of their relationship with their parents) and tend to expand on topics different from those posed by the interviewer [12,13]. A recent review describes the underlying relationship between confidence, low stress, family balance and the improvement of performance at work [14].

The aim of this study is to investigate if there is a relationship between aggression and insecure attachment (avoidance/anxiety) in healthcare workers. Accordingly, two hypotheses (H1-H2) were specified:

H1. We assume that people with an insecure attachment will be more aggressive towards health service users, because according to attachment theory people with anxiety and avoidance have problems in their interpersonal and social relationships.

H2. Given the social stereotypes that exist for men and women, we assume that male professionals will be more aggressive than women.

## Materials and methods

Research design and Procedure

The research was carried out by self-reporting questionnaires, administrated to the participants by the investigator, through Google Forms or health interest sites. Participants volunteered to answer anonymously the two questionnaires, after being informed about the purpose of the research and be assured of the confidentiality and anonymity of their answers. The sample of health professionals in the private and public sectors were selected by the method of random sampling. The survey was conducted from February 2018 to May 2018.

Participants

A sample of 192 health professionals, who were in direct contact/ dealing with patients, has been used (43 men and 149 women), aged 20 to 60 years, in Athens, Greece. Of our sample, 59.9% worked in the public sector, while 24% in the private sector. Still, 19.3% worked in psychiatric hospitals and 37.5% in general.

Measures and instruments

Participants were asked to answer if they had witnessed aggressive behavior in their workplace and if so what it was about, for example, verbal or physical aggression. There was, also, a form in which demographic information was filled in, such as experience, age, work context, gender, educational level and also marital status. The questionnaires administered were the Buss-Perry Aggression Questionnaire [15], in the Greek version [16] and the Adult Attachment Scale [17], in the Greek version [18].

The Aggression Questionnaire is used to measure aggression and is a self-report scale, which consists of 29 items: Participants used a 5-point Likert-type scale (rating from 1: Not at All to 5: Completely) to record the extent to which they thought the items of the questionnaire were characteristic of them. The scale divides aggression into "Physical Aggression" (9 items), "Anger" (7 items), "Hostility" (8 items), and "Verbal Aggression" (5 items). Was used the Adult Attachment Scale [17], in the Greek version [18], which includes 36 questions. The participant is asked to indicate whether he agrees or disagrees with each proposal rating from 1(=strongly disagree) to 7 (=strongly agree). It has two sub-scales: anxiety/obsession and avoidance.

Data analysis

The data collected, coded, recorded and analyzed with the Statistical Package of Social Sciences (SPSS 26) (IBM Corp., Armonk, NY). Descriptive statistics were used for clinical and demographic characteristics of the sample, Pearson r correlation coefficient was used for correlation analyzes and regression analysis was used for effects.

## Results

The research sample consisted of 192 health professionals working in the public and private health sector. The sample consisted of 43 men and 149 women. 24.5% were people aged 20-30, 43.8% 31-40 years old, 26.6% 41-50 years old and 5.2% 51-60 years old. Of the participants, 42.2% were single or in a relationship, 48.4% married and 9.4% divorced. 100 participants (52.1%) were mental health professionals and 92 (47.9%) health professionals of other specialties (Table 1).

**Table 1:** Demographic characteristics of the sample.

Features	n	%
Sex		
Male	43	22.4
Female	149	77.6
Age		
20-30	47	24.5
31-40	84	43.8
41-50	51	26,6
51-60	10	5.2
Education		
Elementary	4	2.1
Post-secondary education	8	4.2
Graduated	83	43.2
Post-graduated	97	50.5
Marital status		
Single	81	42.2
Married	93	48.4
Divorced	18	9.4
Profession		
Mental health professionals	100	52.1
Health professionals of other specialties	92	47.9
Sector		
Public	115	59.9
Private	46	24.0
Other	31	16.1
Type of hospital		
Psychiatric	37	19.3
General	72	37.5
Not in hospital	83	43.2

The reliability and averages of the questionnaires for the overall sample are presented in Table 2:

**Table 2:** Reliability and averages of the scales for the total sample (N = 192).

Questionnaires	Sub-scales	M	SD	Number of questions	Cronbach a
Aggression	Physical aggression	1.6	0.5	9	.69
	Anger	2.1	0.7	7	.81
	Hostility	2.0	0.6	8	.75
	Verbal aggression	2.6	0.6	5	.57
Attachment	Stress	2.9	1.2	18	.92
	Avoidance	2.8	1.0	18	.89

The analysis shows a statistically significant difference in physical aggression between elementary education, post-secondary level, university degree and postgraduate education [F(3,19) = 5.42, p = 0.001]. In order to identify in which educational level there is a significant difference between the two, a post hoc multiple comparison was performed. According to the results of the analysis, the graduates of a post-secondary level (M = 1.9, SD = 0.7) show significantly greater physical aggression than the graduates of elementary level (M = 1.7, SD = 0.5), or the university graduates (M = 1.6, SD = 0.6) or the holders of postgraduate degree (M = 1.5, SD = 0.3). Regarding the other dimensions (anger, hostility and verbal aggression) there were no significant differences between the groups.

There is a statistically significant difference only in physical aggression between men's and women's occupations [t(190) = 2.05, p = 0.042]. According to the results of the analysis, men (M = 1.7, SD = 0.6) show significantly greater physical aggression than women (M = 1.5, SD = 0.4). Regarding the other dimensions of aggression, ie anger, hostility and verbal aggression, no statistically significant differences are observed between men and women (p > 0.05).

There is a statistically significant difference in hostility between employees in a Psychiatric Hospital, in a General Hospital and in private [F(3,188) = 4.29, p = 0.015]. In order to identify which employees demonstrate the significant difference, a post hoc multiple comparison was performed. According to the results of the analysis, the employees in General Hospitals (M = 2.2, SD = 0.7) show significantly more hostility than the employees in Psychiatric Hospitals (M = 1.8, SD = 0.5) or the employees who don’t work in hospitals (M = 2.0, SD = 0.6). Regarding the other dimensions of aggression, for example, anger, physical and verbal aggression, there are no statistically significant differences between the groups (p > 0.05).

There are no statistically significant differences in age and marital status in terms of aggression.

Of the total sample, 73.4% reported that they witnessed aggressive behavior by a health professional in the workplace, namely 20.3% to a colleague, 13.5% to a co-worker and 39.6% to both co-workers and employees. In contrast, 26.6% of the sample did not witness any aggressive behavior in the workplace. The reported cases of aggressive behavior related to verbal aggression at a rate of 50.0%, 3.6% to physical and 19.8% to both verbal and physical aggression (Table 3).

**Table 3:** Witnessing aggressive behavior by a health professional for the total sample (N = 192).

Testimony		Ν	%
Witnessing aggressive behavior	Yes, to a colleague	39	20.3
	Yes, to client	26	13.5
	Both	76	39.6
	No	51	26.6
	Total	192	100.0
The incident concerned	Verbal aggression	96	50.0
	Physical aggression	7	3.6
	Both	38	19.8
	No	51	26.6
	Total	192	100.0

From the analysis of the results (Table 4), it seems that there is a positive correlation of the dimension of avoidance in the type of attachment with physical aggression (r = 0.27, p < 0.001) and hostility (r = 0.30, p < 0.001) while the dimension of stress in the attachment type seems to have a positive correlation with anger (r = 0.26, p < 0.001), hostility (r = 0.49, p < 0.001), physical aggression (r = 0.25, p < 0.001) (Table 4).

**Table 4:** Relationship (Pearson r) between aggression and type of attachment in health professionals. **Correlation is significant at the 0.001 level.  *Correlation is significant at the 0.005 level.

	Anger	Physical aggression	Verbal aggression	Hostility	Avoidance	Stress
Anger						
Physical aggression	0.46**					
Verbal aggression	0.50**	0.25**				
Hostility	0.56**	0.46**	0.30**			
Avoidance	0.14	0.27**	0.12	0.30**		
Stress	0.26**	0.25**	0.11	0.49**	0.54**	

Multiple regression analysis was performed to examine whether the dimensions of stress and avoidance affect anger. The adjusted coefficient of determination R2 is equal to 0.07. The review of regression coefficients found that the dimension of adult attachment stress contributes significantly to the prediction of Anger (β = 0.16, t = 3.0, p = 0.002), while the dimension of avoidance does not contribute (p > 0.05).

Multiple regression analysis was performed to examine whether the dimensions of stress and avoidance affect physical aggression. The adjusted coefficient of determination R2 is equal to 0.11. The review of regression coefficients found that the dimension of adult attachment avoidance contributes significantly to the prediction of physical aggression (β = 0.10, T = 1.42, p = 0.014.) While the dimension of stress does not contribute (p > 0.05). The dimensions of stress and avoidance have no effect on verbal aggression (p > 0.05).

In order to assess whether the dimensions of stress and avoidance have any effect on hostility, the multiple regression analysis was performed. The adjusted coefficient of determination R2 is equal to 0.25. From the review of the regression coefficients, it was found that the dimension of stress contributes significantly to the prediction of Hostility (β = 0.25, t = 6.1, p < 0.001), while the dimension of avoidance does not contribute because the corresponding p > 0.05.

## Discussion

Verbal abuse [19] has been associated with an increased frequency of absenteeism, while disruptive behavior has been shown to adversely affect both job satisfaction and bonding between professionals [6]. The degree of coherence of members of a team, the incompatibility of the goals of individuals or groups, uncertainty, lack of understanding, differences in values, attitudes, needs, beliefs, perceptions and interests are sources of tension and conflict [20].

Disruptive behavior could make communication and collaboration breakdowns among doctors and health professionals. This may provoke medical errors and has an impact on patients’ care quality [21]. Moreover, disruptive behavior has the potential to contact retention issues, effect societal and organization outcomes, patients’ safety and healthcare workers’ well-being [22]. Rosenstein [6], for example, detailed that almost 1/3 of the sample designated that they know of a nurse who had abandoned a hospital as a result of this type of behavior. Another 1/4 said they have heart an example of nurses who had switched schedules, shifts, even departments to circumvent certain doctors. Furthermore, Rosenstein and O’Daniel [23] underlined that staff connections are crucial to healthcare services and that disruptive behavior has an important negative impact. Disruptive behavior could lead to drop of a doctor’s hospital privileges [24] and contributes to litigation risk by promoting patient dissatisfaction [25]. In our work, 73.4% of the sample reported that they have witnessed some kind of aggressive behavior by a health professional in the workplace, namely 20.3% against a colleague, 13.5% against a client and 39.6% to both colleagues and employees. The reported cases involved verbal aggression at a rate of 50.0%, physical aggression at 3.6% and both verbal and physical aggression at 19.8%.

Hazan and Shaver [26] were some of the first researchers who tried to apply attachment theory directly to a job study. They found out that, compared to insecure workers, secure workers enjoyed more of their work, had higher levels of general well-being, and also were less worried about their work relationships. Although, anxious individuals faced the fear of rejection and poor performance and avoidants used the work to avoid social interaction and need to take vacations more than others. Last but not least, a recent review of the literature describes the underlying relationship between secure attachment, effectiveness, low stress, confidence, leadership skills, positive attitudes, good health, family balance, and improved performance [14].

As regards the general population, work relationships are well defined from attachment relationships [27]. A variety of studies in doctors with mental or behavioral problems have recognized high levels of family experiences or an unpleasant childhood. Insecure early attachment experiences can be a risk factor for stress and poor management of stress and problems in medical students and doctors who are exposed to increased demands as caregivers. These findings lead to suggestions for possible research and support interventions. Attachment theory makes specific predictions that may explain why some employees experience burnout or mental fatigue and others do not. Findings from West et al. [28] review suggest that secure attachment is related with lower levels of burnout, the instance that stressfulness is correlated with higher levels and higher avoidance foreseen higher levels of burnout for a variety of variables.

Our analysis shows that the dimension of avoidance has a positive correlation with hostility and physical aggression and the stress dimension has a positive correlation with anger, physical aggression and hostility. Mikulincer and Shaver [10] observed that people with an anxious attachment type, because of their tendency to intensify anxiety and ruminate on unpleasant experiences, are vulnerable to intense and prolonged periods of anger.

The findings of the present study confirm the existence of a positive correlation between the subscales of aggression (anger, physical aggression, verbal aggression, hostility) but also between the dimensions of the attachment type (avoidance, stress). Specifically, there is a positive correlation between anger and physical and verbal aggression and hostility, just as Buss and Perry [15] pointed out, although this finding was described as "unexpected". In contrast, the positive correlation between verbal aggression and hostility with physical aggression, which is also confirmed in the present study, was not considered unexpected by Buss and Perry [15]. The dimension of stress in the attachment type has a positive correlation with the dimension of avoidance, something that was also observed in the study of Sibley et al. [29].

According to the results of the analysis, men and graduates of post-secondary education show the greatest physical aggression. Also, General Hospital staff show the greatest hostility. There was no significant difference identified between the mental health professionals and health professionals of other specialty. Therefore, from the above findings both of our hypotheses are confirmed.

Our study naturally presented some limitations worth mentioning. Firstly, further research is needed to generalize the results with a larger sample of healthcare workers. Secondly, it would be good in future research to be used a larger numerical sample of doctors who work in hospitals and see more acute cases, in order to measure the existence of aggression towards patients. Furthermore, given the established relationship between gender and specific forms of violence, the gender skew in this sample is a significant limitation. Moreover, the aggression questionnaire measures various situations of aggression and is not a questionnaire that measures the aggression of hospital staff.

## Conclusions

This research examines the relationship between adult attachment and self-reported aggression in healthcare professionals. There is already enough literature on the link between attachment and anger/aggression in the general population. Although, this study aims to extend these results to healthcare professionals. The general estimate is that a quarter to a third of researchers and academics are dealing with such behaviors each year, a much higher rate than other professions. No one can say for sure if or to what extent the situation is getting worse from year to year. It is important that violence in the workplace is recognized as an underlying occupational risk and not just as a matter of criminal law. More research is needed to study the phenomenon in order to make it more understandable, to investigate the causes that cause it and to maintain it, as well as to formulate proposals and measures for its prevention, avoidance and treatment. Managers need to be aware of the risk factors for violence in the workplace and take steps to prevent violent incidents.
